# Where are we now? Emerging opportunities and challenges in the management of secondary hyperparathyroidism in patients with non-dialysis chronic kidney disease

**DOI:** 10.1007/s40620-021-01082-2

**Published:** 2021-06-25

**Authors:** Markus Ketteler, Patrice Ambühl

**Affiliations:** 1 Allgemeine Innere Medizin und Nephrologie, Robert-Bosch-Krankenhaus, Stuttgart, Germany; 2 Institut für Nephrologie, Stadtspital Waid und Triemli Zürich, Zurich, Switzerland

**Keywords:** Secondary hyperparathyroidism, Non-dialysis chronic kidney disease, Parathyroid hormone, Vitamin D, Extended-release calcifediol

## Abstract

**Abstract:**

Rising levels of parathyroid hormone (PTH) are common in patients with chronic kidney disease (CKD) not on dialysis and are associated with an elevated risk of morbidity (including progression to dialysis) and mortality. However, there are several challenges for the clinical management of secondary hyperparathyroidism (SHPT) in this population. While no recognised target level for PTH currently exists, it is accepted that patients with non-dialysis CKD should receive early and regular monitoring of PTH from CKD stage G3a. However, studies indicate that adherence to monitoring recommendations in non-dialysis CKD may be suboptimal. SHPT is linked to vitamin D [25(OH)D] insufficiency in non-dialysis CKD, and correction of low 25(OH)D levels is a recognised management approach. A second challenge is that target 25(OH)D levels are unclear in this population, with recent evidence suggesting that the level of 25(OH)D above which suppression of PTH progressively diminishes may be considerably higher than that recommended for the general population. Few therapeutic agents are licensed for use in non-dialysis CKD patients with SHPT and optimal management remains controversial. Novel approaches include the development of calcifediol in an extended-release formulation, which has been shown to increase 25(OH)D gradually and provide a physiologically-regulated increase in 1,25(OH)_2_D that can reliably lower PTH in CKD stage G3–G4 without clinically meaningful increases in serum calcium and phosphate levels. Additional studies would be beneficial to assess the comparative effects of available treatments, and to more clearly elucidate the overall benefits of lowering PTH in non-dialysis CKD, particularly in terms of hard clinical outcomes.

**Graphic abstract:**

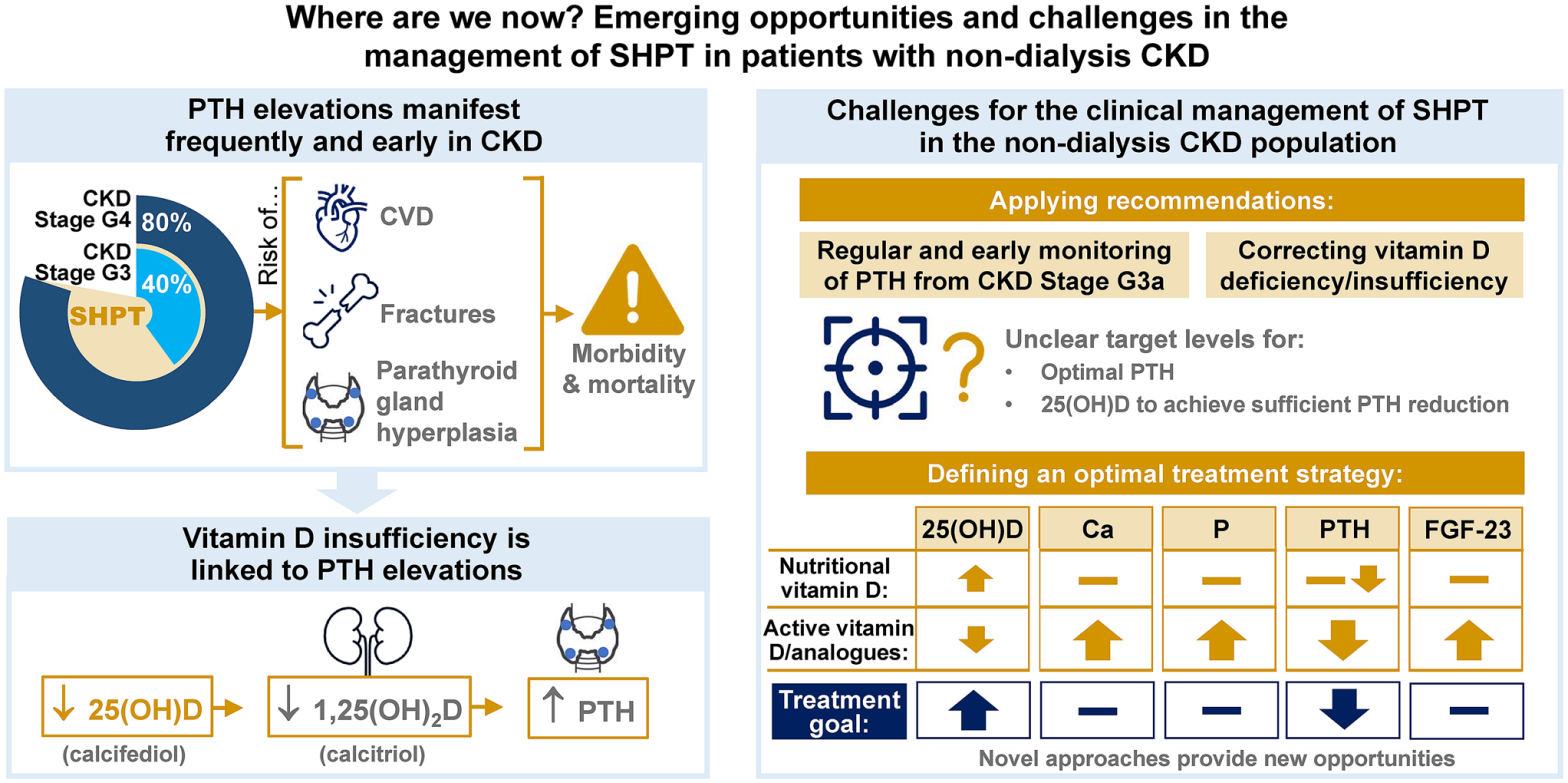

## Introduction

Chronic kidney disease (CKD) is a major and growing global public health burden that is associated with significant morbidity and has continued to rise in rank among the leading causes of death over the last 3 decades [1]. Progression of CKD is associated with increasing risk of death, cardiovascular events, and hospitalisation [2, 3]. In 2017, CKD was estimated to affect 9.1% of the global population; however, only a minority had advanced renal dysfunction. CKD and its effect on cardiovascular disease were estimated to have resulted in 2.6 million deaths and 35.8 million disability-adjusted life years [1]. By 2040, CKD is predicted to be the fifth leading cause of years of life lost globally [4].

Complex mineral metabolism disturbances and loss of homeostasis are common in CKD and are associated with declining kidney function. Recent findings from an end-stage kidney disease longitudinal analysis of the Chronic Renal Insufficiency Cohort Study (n = 847) found that abnormalities in mineral metabolism intensified approximately 5 years before end-stage kidney disease, or at CKD stage G3 [5].

A common and early complication of CKD is secondary hyperparathyroidism (SHPT), characterised by elevated serum parathyroid hormone (PTH) and parathyroid hyperplasia, that develops as a consequence of the mineral metabolism disturbances of several biochemical parameters (including increases in fibroblast growth factor-23 [FGF-23], and reductions in 25-hydroxyvitamin D [25(OH)D] and 1,25-dihydroxyvitamin D [1,25(OH)_2_D], and hypocalcaemia and hyperphosphataemia) [6–9]. A review of the pathogenesis of SHPT in CKD is beyond the scope of this article, and the reader is referred to review articles on this subject (e.g., Cunningham, 2011) [6]. The characteristic mineral metabolism disturbances and rising PTH levels of SHPT independently predict risk of fractures, vascular events, progression to dialysis and death [2, 3, 10, 11]. As such, approaches to manage SHPT have formed an important focus of treatment in CKD. Use of calcimimetics, calcitriol, and/or active vitamin D analogues (alone and in combination) has been the mainstay of treatment of SHPT for patients on haemodialysis for decades (targeting PTH levels of 2–9 × upper limit of normal), with parathyroidectomy remaining a valid treatment option, especially in cases when PTH-lowering therapies fail [7].

By contrast, the optimum management of SHPT treatment in non-dialysis CKD is not as clearly understood. For example, as reflected in recent guidelines, studies have called into question the routine use of calcitriol and active vitamin D analogues for the management of SHPT in CKD stage G3a–G5 due to increased risk of hypercalcaemia [7]. Studies also indicate that, at variance with the dialysis setting, knowledge amongst physicians about mineral and bone disorder management in non-dialysis CKD is scarce [12]. However, recently published data, particularly regarding the role of vitamin D, alongside new therapeutic advances, are highly relevant and offer new insights into the management of SHPT in non-dialysis CKD. In light of emerging evidence, the aim of this review is to reassess the opportunities and challenges in the management of SHPT in patients with non-dialysis CKD specifically, with a focus on the role of vitamin D.

## What’s new? Insights into the rationale for SHPT and elevated PTH as a therapeutic target in non-dialysis CKD

While the adverse effects of SHPT are well recognised in CKD patients on dialysis (stage G5D), elevations in PTH characteristic of SHPT manifest frequently in non-dialysis CKD and from as early as CKD stage G2 [13]. SHPT (PTH > 65 pg/mL) affects approximately 40% of patients with CKD stage G3 (with the percentage rising from stage G3a–G3b), rising to approximately 80% in CKD stage G4 [14]. Recent studies demonstrate that SHPT is associated with the risk of cardiovascular events regardless of CKD stage [11], and in patients with non-dialysis CKD, PTH is a predictor of risk of fractures, vascular events, progression to dialysis and death [2, 15].

**Fig. 1:**
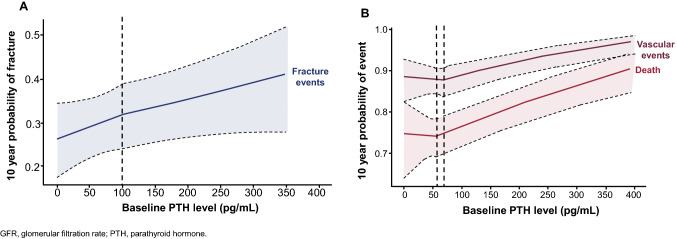
PTH levels independently predict **A** fracture, and **B** vascular events, and death in CKD Stage G3–G4 (Reprinted by permission from Springer Nature. Adapted from Geng S, et al. Osteoporos Int 2019;30:2019–2025 [2]). **A** Ten-year probability of fractures, based on baseline PTH levels. The hazards of fracture rose steadily with increasing PTH values. No significant difference was noted between the slope of the curve for fracture risk, when comparing odds of fracture in subjects with baseline PTH levels above and below a PTH value of 101 pg/mL. **B** Ten-year probability of vascular events and death, based on baseline PTH levels. The figure demonstrates that hazards of vascular events and death were lowest at PTH values of 69 and 59 ng/mL, respectively

In an analysis by Geng et al. [2] of electronic health records (between 1985 and 2013) from over 5000 adults with baseline CKD stage G3–G4 (mean follow-up of 23 ± 10 years), PTH was found to be an independent predictor of fracture, vascular events, and death (Fig. 1). The risk of vascular events and death were lowest when baseline PTH levels were 69 and 58 pg/mL, respectively. However, unlike vascular events and death, no baseline threshold of PTH was identified for fracture risk, and the risk of fracture continued to rise in parallel with rising PTH [2]. A recent multicentre prospective cohort study from the Fukuoka Kidney Disease Registry (3,384 non-dialysis CKD patients) explored the relationship between PTH concentrations and the prevalence of atrial fibrillation. PTH was evaluated as a potential risk factor and assessed in quartiles (Q1 5–46, Q2 47–66, Q3 67–108, Q4 109–1660 pg/mL). Higher PTH concentrations (Q2–Q4) were significantly and incrementally associated with an increased prevalence of atrial fibrillation in this patient group. Using Q1 as the reference group the adjusted odds ratios for the prevalence of atrial fibrillation were 1.33 (0.76–2.34), 1.82 (1.06–3.13), and 1.99 (1.08–3.64), for Q2–Q4, respectively (P = 0.016) [15].

Untreated, SHPT results in continually increasing PTH levels. In randomised controlled trials of patients with non-dialysis CKD, PTH levels continued to increase in placebo-treated or untreated patients over the duration of the studies [16–19]. New data from the Dialysis Outcomes and Practice Patterns Study (DOPPS) highlight that elevated PTH > 600 pg/mL prior to haemodialysis is strongly associated with uncontrolled PTH during haemodialysis, despite more aggressive SHPT treatment at that time. High PTH levels during the first year of haemodialysis were, in part, suggested to be reflective of suboptimal pre-dialysis SHPT management [20]. In addition, a recent retrospective analysis of 13,772 incident haemodialysis patients demonstrated that PTH levels of ≥ 250 pg/mL were independently associated with a more rapid decline in residual kidney function; however, higher PTH levels may have just reflected progressively impaired kidney function [10]. In renal transplant patients, elevated PTH levels pre-transplant have been shown to be independently associated with a significant risk for graft failure censored for death [21], as well as being a risk factor for post-transplant nephrocalcinosis [22]. Parathyroidectomy in post-transplant patients has also been associated with acute graft failure [23]. Data such as these suggest that the effectiveness of an intervention decreases as CKD progresses. Indeed, analysis of the Chronic Renal Insufficiency Cohort Study cohort (n = 3683) followed patients with CKD stage G2–G4 over a median of 9.5 years, and revealed patients spent progressively less time in each successive stage of CKD, with a median of 7.9, 5.0, 4.2, and 0.8 years in CKD stages G3a, G3b, G4, and G5, respectively [24]. Parathyroid hyperplasia and sustained elevations in PTH with SHPT progression due to delayed treatment are accompanied by progressive reductions in sensitivity to calcium and vitamin D regulation [6] and therefore a risk of treatment resistance later in the disease course. Parathyroidectomy may need to be considered if patients become unresponsive to SHPT treatment, have persistently elevated PTH levels, and refractory hypercalcaemia or hyperphosphataemia [6, 25]. However, parathyroidectomy can be associated with post-surgical complications, including severe hypocalcaemia [25].

Although optimal PTH levels for patients with CKD stage G3a to G5 are not clearly defined, the potential adverse consequences of prolonged PTH elevations are reflected by the fact that the Kidney Disease: Improving Global Outcomes (KDIGO) CKD-mineral and bone disorder (CKD-MBD) guidelines recommend regular monitoring of PTH levels starting in CKD stage G3a, in order to identify patients with progressively rising or persistently elevated PTH levels above the upper limit of normal, so that at-risk individuals can be recognised and evaluated for modifiable risk factors [7]. However, studies indicate that adherence to mineral and bone disorder monitoring recommendations in non-dialysis CKD may be suboptimal, and that competing priorities in CKD may frequently distract from regular monitoring of mineral and bone disorder in these patients [12, 26]. A large prospective cohort study from the Chronic Kidney Disease Outcomes and Practice Patterns Study [CKDOPPS] involving 7658 patients with CKD also identified significant variations in upper target PTH levels among nephrologists [27].

## Vitamin D in the management of SHPT in non-dialysis CKD

Vitamin D insufficiency is highly prevalent among patients with CKD, being more common than in the general population [28, 29] and affecting 71–84% of patients with CKD stage G3–G4, respectively (insufficiency defined in the study as ≤ 75 nmol/L; ≤ 30 ng/mL) [29]. Low levels of vitamin D are independently associated with an increased risk of CKD progression, morbidity and mortality in non-dialysis CKD [30]. Low levels of vitamin D are also frequently linked to elevations in PTH in non-dialysis CKD as indicated by early data from 3488 patients enrolled in the CKDOPPS, a prospective cohort study of patients with estimated glomerular filtration rate (eGFR) < 60 mL/min/1.73 m^2^ from national samples of nephrology clinics in Brazil, France, Germany and the US [31]. These data reflect the prominent role of vitamin D in the pathogenesis of SHPT.

**Fig. 2:**
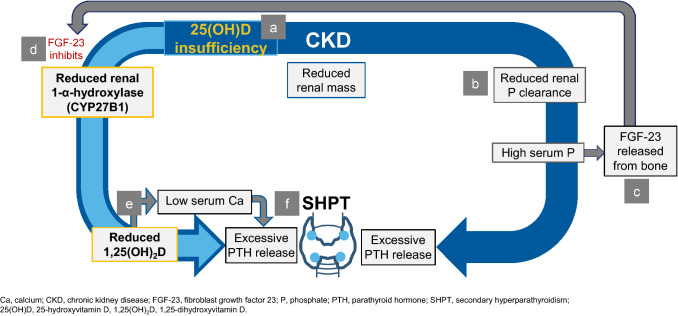
Role of Vitamin D in SHPT pathophysiology [6, 14]. **a** Vitamin D insufficiency stimulates PTH release which is exacerbated by renal impairment and impaired synthesis of active vitamin D in the kidney. **b** Declining glomerular filtration rate as CKD progresses leads to reduced phosphate clearance, increasing serum phosphate. **c** In response, FGF-23 is released from the bone. **d** FGF-23 down regulates CYP27B1 reducing renal 1-α-hydroxylase. Elevations in serum FGF-23 in CKD also lead to up-regulation of 25(OH)D and 1,25(OH)_2_D catabolism. **e** This leads to a decline in 25(OH)D and 1,25(OH)_2_D production, and as a result, reduced serum calcium levels. **f** Hypocalcaemia and 1,25(OH)_2_D deficiency in CKD patients result in the excessive PTH secretion and parathyroid gland hyperplasia that characterise SHPT

Vitamin D has an important physiological role for tissue homeostatic mechanisms, including potentially pleiotropic effects [32]. In the setting of normal kidney function, low levels of vitamin D are detected by the parathyroid glands, with a consequent increase in the production and release of PTH [6, 33]. In the setting of CKD, these elevations in PTH are part of an adaptive process that gradually become maladaptive in response to declining kidney function, causing abnormalities in several biochemical parameters including impaired phosphate excretion, increased FGF-23, hypocalcaemia and failure to bioactivate vitamin D; the combined effect of these multiple pathways is to promote the progression of SHPT as detailed in Fig. 2 [6].

The known pathophysiology, together with recent data, illustrate the rationale for treatment of SHPT and vitamin D insufficiency/deficiency in non-dialysis CKD, and the KDIGO guidelines for the management of CKD-mineral and bone disorder recommend that patients with CKD stage G3–G4 and progressively rising or persistently elevated PTH levels above the upper limit of normal should be evaluated for vitamin D deficiency as one of the modifiable risk factors [7]. Other modifiable risk factors in this context include hyperphosphataemia and hypocalcaemia [7]. In the setting of CKD stage G3a–G5D, 25(OH)D levels might be measured, with repeated testing depending on baseline values and therapeutic interventions; however, as previously noted, adherence to CKD-mineral and bone disorder monitoring recommendations may be suboptimal [12, 26]. Vitamin D deficiency/insufficiency should be corrected using recommended treatment strategies [7, 28, 34].

## Exploring the benefits of current and emerging approaches for the management of SHPT in non-dialysis CKD

**Fig. 3:**
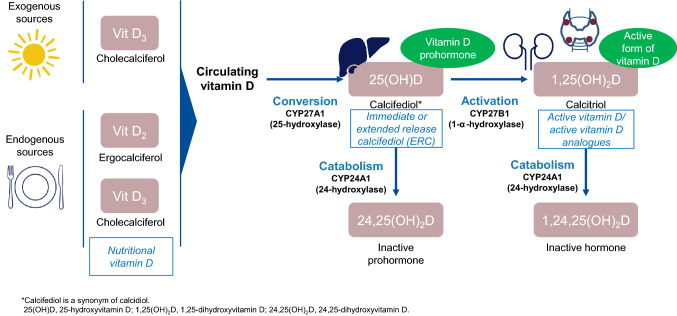
Vitamin D metabolism [35, 43]. Vitamin D3 is generated in the skin under the influence of UV-B radiation, vitamin D2 and D3 are obtained from dietary sources by absorption through the intestine. In the liver, circulating vitamin D is converted to 25(OH)D by 25-hydroxylase (CYP2RI, CYP27A1). 25(OH)D is then converted to the active vitamin D metabolite, 1,25(OH)_2_D in the kidney and in extra-renal locations such as the parathyroid gland by the enzyme 1-α-hydroxylase (CYP27B1). 1-α-hydroxylase is regulated by several negative feedback mechanisms, including PTH, calcitonin, and FGF-23. Active vitamin D can be deactivated by 24-hydroxylase. The activity of 24-hydroxylase (CYP24A1) to degrade 25(OH)D and 1,25(OH)_2_D is governed by these negative feedback loops, and is a key determinant of the net amount of active vitamin D. Blue boxes represent where the therapeutic forms of vitamin D enter the metabolic pathway

The term vitamin D represents native or nutritional vitamin D, these include both vitamin D2 (ergocalciferol) and vitamin D3 (cholecalciferol). Both vitamin D2 and D3 are hydroxylated in the liver (by the cytochrome P450 enzymes CYP2R1, CYP27A1) to calcifediol [25(OH)D]. The conversion to calcitriol [1,25(OH)_2_D; the active form of vitamin D] then occurs via 1-α-hydroxylation (by the cytochrome P450 enzyme CYP27B1) mainly in the kidney, but also at other extrarenal sites such as the parathyroid glands. Active vitamin D is then catabolised to its biologically inert forms (Fig. 3) [33, 35, 36].

In patients with renal impairment, levels of both 25(OH)D and 1,25(OH)_2_D are reduced as CKD progresses, with active vitamin D reduced not only due to impaired synthesis in the kidney, but also as a result of the down-regulation of 1-α-hydroxylase by serum FGF-23, which becomes elevated in response to an increased phosphate balance. Indeed, studies have suggested that the efficiency of vitamin D hydroxylation declines with declining renal function [37, 38]. Elevations in serum FGF-23 in CKD also lead to up-regulation of 25(OH)D and 1,25(OH)_2_D catabolism via the cytochrome P450 enzyme CYP24A1, leading to vitamin D inactivation [6, 39]. Extra-renal activation of 25(OH)D to 1,25(OH)_2_D may play an important role in active vitamin D production among CKD patients in whom renal function is impaired [35, 40].

The therapeutic forms of vitamin D available have activity at different points in the metabolic pathway (Fig. 3). They include nutritional vitamin D (e.g., cholecalciferol, ergocalciferol), vitamin D prohormone (e.g., calcifediol) and active vitamin D/active analogues (e.g., calcitriol, paricalcitol).

### Nutritional vitamin D

Nutritional vitamin D supplements are available as vitamin D2 (ergocalciferol) and vitamin D3 (cholecalciferol). Cholecalciferol has been shown to be more effective in elevating and maintaining serum 25(OH)D levels in healthy adults than ergocalciferol at equimolar doses, with a longer half-life [41]. The half-lives of ergocalciferol and cholecalciferol are affected by vitamin D binding protein concentration and genotype [28]. In one study of healthy men (n = 36) the mean half-life of ergocalciferol was 13.9 (2.6) days, significantly shorter than cholecalciferol (15.1 [3.1] days; p = 0.001) [42].

Nutritional vitamin D supplements are not specifically indicated for SHPT in non-dialysis CKD, and while many studies have explored their therapeutic potential, the evidence supporting a positive impact on serum 25(OH)D and PTH in non-dialysis CKD, has been largely based on data extrapolated from observational studies in patients with CKD stage G3–G5D and renal transplant recipients with mixed results [44].

Indeed, more recent studies suggest that nutritional vitamin D supplements do not consistently and reliably lower PTH in non-dialysis CKD patients, even at higher doses [45, 46]. A 2016 meta-analysis of non-dialysis CKD patients treated with nutritional vitamin D (cholecalciferol or ergocalciferol) versus placebo across four randomised controlled trials demonstrated that although nutritional vitamin D increased 25(OH)D levels and lowered PTH when compared to placebo, data were based on a small population of 122 patients and there was substantial heterogeneity in effect sizes between studies; it therefore concluded that additional data were needed [45, 46]. In a study of patients with non-dialysis CKD (n = 95), high-dose (8000 IU/day) cholecalciferol was shown to increase calcitriol [1,25(OH)_2_D] levels, and although further increases in PTH were not seen in the cholecalciferol group, which may have to be regarded as a “partial response”, PTH levels were not reduced from baseline and the proportion of patients achieving a 30% decrease in PTH levels did not differ from placebo [16]. In a subsequent 2020 meta-analysis of non-dialysis CKD patients treated with nutritional vitamin D (cholecalciferol or ergocalciferol) across 14 randomised controlled trials (N = 745) only a small reduction in PTH was observed from baseline in nutritional vitamin D-treated patients [17]. Changes in PTH relative to placebo or untreated patients appear to be driven by PTH increases in the comparator groups rather than decreases in the treatment group, with substantial heterogeneity in effect sizes again observed between studies [16, 17]. The complex and variable nature of nutritional vitamin D absorption, distribution and activation may reduce its effect on 25(OH)D levels and contribute to its limited ability to reduce PTH [34]. Nutritional vitamin D also has a propensity to be deposited in adipose tissue due to its lipophilic properties, and this mechanism likely plays a significant role in reducing the amount of nutritional vitamin D that can be presented to the liver for conversion to 25(OH)D [43, 47]. Indeed, vitamin D insufficiency is common in obese individuals; studies have shown that low levels of serum 25(OH)D can fail to recover after nutritional vitamin D supplementation in these subjects [48]. Together these effects may mitigate the impact of vitamin D supplementation on available levels of active vitamin D.

### Immediate-release calcifediol

The potential ability of immediate-release calcifediol to reduce serum PTH has long been recognised, although treatment was associated with increases in serum calcium and phosphate and early results were variable [49, 50]. Calcifediol is readily absorbed and results in a more rapid increase in serum 25(OH)D compared to oral cholecalciferol [51]. Based on the results of nine randomised controlled trials comparing cholecalciferol with calcifediol, calcifediol was over three times more potent than cholecalciferol [51]. However, immediate-release calcifediol is not indicated for SHPT and is not able to provide clinically meaningful reductions in PTH in CKD patients [40]. Immediate-release calcifediol produces abrupt and sudden increases in serum 25(OH)D and/or 1,25(OH)_2_D, and although levels increase initially, the pharmacological ‘surges’ that subsequently occur may trigger down-regulation of CYP27B1 (limiting further conversion of 25(OH)D to 1,25(OH)_2_D) and/or stimulating up-regulation of 25(OH)D and 1,25(OH)_2_D catabolism via CYP24A1 leading to vitamin D inactivation, and subsequently little change in plasma PTH [39, 40, 47, 52].

### Active vitamin D/analogues

Active vitamin D including calcitriol, and active vitamin D analogues including paricalcitol and alfacalcidol, are variously indicated for the prevention and/or treatment of SHPT in non-dialysis CKD, although licensing and availability differs between countries [40]. Active vitamin D and its analogues suppress PTH [53], however, their mechanism of action bypasses the physiological regulation of vitamin D metabolism [40]. Active vitamin D and its analogues may, therefore, also lead to surges of 1,25(OH)_2_D following administration, which can induce vitamin D catabolism via CYP24A1 (24-hydroxylase), causing excessive increases in 24,25(OH)D3 and 1,24,25(OH)D3, respectively. Importantly, active vitamin D and its analogues are also associated with an increased risk of hypercalcaemia and risk of accelerated vascular calcification [54–56]. Indeed, recent studies of CKD stage G3–G4 patients with SHPT treated with paricalcitol (PRIMO and OPERA studies) failed to demonstrate improvements in hard outcomes (left ventricular mass and function) but found an increase in the risk of hypercalcaemia [57, 58]. In PRIMO, hypercalcaemia occurred in 22.6% of patients and was the main reason given for study withdrawal [57]. The OPERA study reported a higher incidence of hypercalcaemia (43.3%) despite the use of a lower daily dose of paricalcitol (1 μg/day), although concomitant use of calcium-based phosphate binders was noted in a high proportion of patients [58]. In a recent meta-analysis of six randomised controlled trials in 799 non-dialysis CKD patients treated with paricalcitol or alfacalcidol versus placebo, the PRIMO and OPERA studies accounted for a large proportion of the observed episodes of hypercalcaemia; however, even when they were excluded in a sensitivity analysis, there was still a significantly increased risk of hypercalcaemia in patients treated with active vitamin D or its analogues versus placebo [54]. This risk of hypercalcaemia prompted a re-evaluation of the risk–benefit profile of these agents in non-dialysis CKD, and guidelines no longer recommend routine use of calcitriol or vitamin D analogues in patients with CKD stage G3a–G5 [7]. Participants in the PRIMO and OPERA trials had moderately increased PTH levels, which were potentially ‘overcorrected’, thus therapy with vitamin D analogues may be reserved for patients with CKD stage G4–G5 with progressive and severe SHPT [7].

### Extended-release calcifediol

Extended-release calcifediol (ERC; EU term: prolonged-release calcifediol) is an orally administered prohormone of active vitamin 1,25(OH)_2_D3 in a prolonged release formulation. The formulation of ERC confers unique pharmacokinetic properties and means there is a slow and steady release of calcifediol over an extended 12-h period [52, 59]. As a consequence, ERC does not trigger the rapid surges of 25(OH)D and 1,25(OH)_2_D levels seen with immediate-release calcifediol, thus avoiding the subsequent triggering of the negative feedback loop discussed above [52] and enabling increases in 1,25(OH)_2_D that are physiologically controlled [59]. 1,25(OH)_2_D binds to the vitamin D receptor in target tissues and activates vitamin D-responsive pathways, leading to reduced PTH synthesis and secretion by the parathyroid glands [59].

The efficacy and safety of oral ERC in patients with CKD stage G3–G4 was demonstrated in two Phase 3 clinical trials [19, 59]. In these studies, 429 patients with CKD stage G3–G4, SHPT and vitamin D insufficiency were treated with 30 µg ERC or placebo daily for 12 weeks, 30 or 60 µg ERC or placebo for 14 weeks then 30 or 60 µg ERC for up to 52 weeks (extension study). A steady increase in serum 25(OH)D levels was seen in both studies (p < 0.0001 versus placebo), with 33% and 34% of patients in each study achieving the primary endpoint of a ≥ 30% reduction in PTH from baseline at Week 26 (versus 8% and 7%, respectively, with placebo) [19, 59]. In the open-label extension phase of the trial, patients who were switched from placebo to ERC experienced a decline in plasma PTH levels at a similar rate to those seen with active treatment in the blinded studies. For those patients who continued on ERC through the randomised and open-label phase, the gradual decreases in plasma PTH continued and were maintained over one year of therapy. A further analysis of data revealed that ERC produced exposure-dependent reductions in plasma PTH and bone turnover markers at mean serum total 25(OH)D levels ≥ 50 ng/mL [60]. In addition, plasma PTH levels were progressively suppressed with higher serum total 25(OH)D levels, regardless of CKD stage. Gradual elevation of mean serum 25(OH)D with ERC to levels as high as 92.5 ng/mL over a 52-week period did not increase mean serum 1,25(OH)_2_D levels above the upper limit of normal (62 pg/mL) [60]. These findings support the hypothesis that 25(OH)D can be activated extra-renally by CYP27B1 in parathyroid and many other tissues. Declining kidney function and its resultant effect on declining expression of renal CYP27B1 did not seem to lead to less conversion of 25(OH)D to 1,25(OH)_2_D [59, 60]. Changes in plasma PTH versus baseline were significant at the end of treatment (p < 0.05) for subjects with 25(OH)D ≥ 50.8 ng/mL. It should be noted that, for subjects with 25(OH)D ≥ 50.8 ng/mL, reductions in PTH appeared to attenuate as mean serum total 25(OH)D approached the highest levels (92.5 ng/mL) [60].

Treatment-emergent adverse events were comparable between the treatment and placebo arms of the ERC Phase 3 trials, with minimal changes in serum calcium and phosphate, and hence a low risk for hypercalcaemia and hyperphosphataemia. Gradual elevation of 25(OH)D with ERC to levels as high as 92.5 ng/mL (231.3 nmol/L) over a 26-week period had no adverse effects on safety parameters, and mean serum 1,25(OH)_2_D levels did not increase above the upper limit of normal (62 pg/mL) [59].

Emerging real-world data supports the tolerability and effectiveness of ERC in routine clinical practice. Recent retrospective analyses of medical chart data from 18 US nephrology clinics included patients with CKD stage G3–G4, a history of SHPT and vitamin D insufficiency, who received different interventions including ERC (n = 174), active vitamin D or its analogues (n = 55) and nutritional vitamin D (n = 147). Serum 25(OH)D levels of ≥ 30 ng/mL were achieved by approximately 70% of patients, with about 40% achieving a ≥ 30% reduction in PTH—similar values to those seen in clinical trials, despite higher baseline PTH levels and the use of a lower daily ERC dose [61]. In the same dataset, patients treated with active vitamin D analogues had a small, but statistically significant increase in serum calcium levels, which was not seen with ERC or nutritional vitamin D. In addition, nutritional vitamin D was more commonly used in less severe CKD (69% stage G3 versus 31% stage G4) while ERC and active vitamin D were used to treat more severe CKD (ERC used in 46% stage G3 versus 53% stage G4, and active vitamin D in 38% stage G3 versus 62% stage G4) [62].

### Other potential therapeutic options in non-dialysis CKD

Calcimimetics act by suppressing PTH secretion through activation of the parathyroid calcium-sensing receptor or amplification of the glands’ sensitivity to extracellular ionised calcium [63, 64]. While demonstrated to be highly effective in reducing PTH levels, calcimimetics are only indicated for CKD patients on haemodialysis, with studies of these agents in CKD Stage G3–G4 showing an increased risk of hypocalcaemia and hyperphosphataemia in these patients [7, 64, 65].

As discussed previously, parathyroidectomy can be a highly effective treatment for SHPT, but is associated with a risk of severe hypocalcaemia, and potentially, persistence or recurrence of SHPT due to residual or autotransplanted parathyroid tissue [6, 25]. There is also evidence that, at least in patients with CKD stage G5D, parathyroidectomy carries with it significant risks of morbidity, hospitalisation and mortality, predominantly related to sepsis and acute coronary syndrome [66]. Guidelines therefore suggest parathyroidectomy be reserved for patients with CKD stage G3a–G5D and severe SHPT which is resistant to medical or pharmacological therapy [7].

## What are the current clinical challenges in the management of SHPT in non-dialysis CKD?

Despite advances in our understanding, the optimal management of SHPT in non-dialysis CKD is challenging in clinical practice. The difficulties around lack of data to support clinical decision-making are acknowledged by the most recent KDIGO CKD mineral and bone disorder guideline update, which states that despite the recent completion of key clinical trials “large gaps of knowledge still remained” [7, 67]. We consider here four key questions within the context of recently published data.

### How should we identify patients with non-dialysis CKD suitable for treatment of SHPT in clinical practice?

While PTH measurement is recognised as being very important for the follow-up of patients with CKD, insight on such measurement and its clinical relevance in non-dialysis CKD continues to evolve [9]. Modest increases in PTH may represent an appropriate adaptive response to declining kidney function due to phosphaturic effects and increasing bone resistance to PTH [8] and there remains an absence of clinical data from which to derive thresholds above which PTH levels should be considered maladaptive and at which treatment should therefore be initiated. However, regular monitoring and treatment of underlying modifiable risk factors (such as vitamin D deficiency) may help determine adaptive versus maladaptive changes. Guideline recommendations have therefore been revised to reflect the transition of the parathyroid to a maladaptive response, with the recommendation to identify patients with PTH levels ‘persistently’ above the upper limit of normal (65 pg/mL) and ‘progressively rising’, emphasising that treatment of SHPT should not be initiated in response to a single elevated value but should be based on trends [8]. Current guidelines recommend regular monitoring of PTH in patients with non-dialysis CKD from CKD stage G3a, in order to identify these individuals, with monitoring intervals based on baseline PTH levels and CKD progression [7]. However, despite recommendations, studies indicate that knowledge of CKD-mineral and bone disorder management in non-dialysis CKD may be scarce and that competing priorities in CKD, such as management of comorbid disease, can frequently distract from CKD-mineral and bone disorder monitoring in non-dialysis CKD patients [12, 26]. For example, a large study following 799,300 patients with CKD Stage G3–G5 concluded that laboratory testing for CKD-mineral and bone disorder biochemical markers was suboptimal in relation to KDIGO guidelines [12].

Further evaluations to assess the possible impact of persistent PTH elevations, for example, by bone density testing (dual energy X-ray absorptiometry scan), may help to identify the presence of pathologically relevant effects of persistently elevated or progressively rising PTH. While bone densitometry does not distinguish between high and low bone turnover, the gold standard for making this distinction is a bone biopsy, which is both invasive and difficult, so it is not routinely performed, particularly in the setting of high PTH levels [68]. Measurement of markers such as bone-specific alkaline phosphatase could potentially identify patients with increased bone turnover. Bone-specific alkaline phosphatase is essential for biomineralisation, and recent findings also demonstrate that it has a crucial role in the pathogenesis of vascular calcification, identifying it as a promising predictor of mortality in CKD [69].

While not currently available for non-dialysis CKD, for patients with CKD on dialysis there are established criteria for assessing patients with ‘unclear’ significance of SHPT. An integrated approach in dialysis patients may include measurement of bone turnover markers, such as bone-specific alkaline phosphatase. In dialysis patients with very ‘low’ or very ‘high’ PTH, bone-specific alkaline phosphatase measures could be helpful to better differentiate the type of bone disease (low versus high turnover). In dialysis patients with intermediate PTH and bone-specific alkaline phosphatase, a bone biopsy may be necessary to diagnose the type of bone disorder. However, it is anticipated that non-dialysis CKD patients with such changes are likely to be relatively rare, and no such approaches are currently available for this patient group or are not easily implemented into routine management for SHPT.

### What levels of PTH should we be aiming for following treatment of SHPT in non-dialysis CKD?

While recommended target levels for PTH in dialysis patients (2–9 × upper limit of normal) have been set out in treatment guidelines, similar targets for non-dialysis CKD patients are unclear for the reasons outlined above [7]. The clinical endpoint most frequently used in clinical trials of SHPT in non-dialysis CKD is ≥ 30% reduction in PTH from pre-treatment baseline levels [18, 19, 70, 71]. While this was agreed with regulatory bodies to be the best available clinical and biological marker to determine a statistically significant change from baseline, studies using this endpoint cannot offer further insight into the specific PTH target levels we should be aiming for in this population. In addition, there is a lack of data linking the achievement of specific PTH levels following treatment intervention with hard outcomes (for example fracture risk and cardiovascular disease) in non-dialysis CKD. There are of course recognised challenges associated with designing trials that provide conclusive results for such endpoints in patients with a progressive and complex disease like CKD. For example, clinical studies of the duration required are not always feasible in a progressive disease like CKD, as patients might require additional treatments such as dialysis, which could confound the results. Studies assessing the impact of SHPT treatment on surrogate endpoints for cardiovascular risk have been performed in an effort to overcome these challenges. However, the PRIMO and OPERA trials of paricalcitol treatment of SHPT in CKD stage G3–G4 did not identify any significant differences between the active and placebo arms in terms of surrogate endpoints of cardiovascular risk (left ventricular mass index—an intermediate endpoint for cardiovascular events), although there were fewer cardiovascular-related hospitalisations in the paricalcitol versus placebo arms [57, 58]. Factors such as sample size, study duration and baseline imbalances between the randomised groups are thought to have potentially impacted the results [57, 58]. Novel surrogates for hard outcomes are gaining support and offer a potential avenue to gain further insight into the potential benefits of PTH reduction in non-dialysis CKD. One surrogate gaining interest in recent years is the T50 test, a blood test that has been developed to determine the calcification propensity in blood [72]. Vascular calcification is frequently observed at high rates in patients with CKD and may be a central mediator of cardiovascular sequelae [73]. The T50 test provides an estimate of the efficiency of an individual’s anticalcification system to inhibit the formation of calcium phosphate nanocrystals [74]. A shorter serum T50 (i.e., accelerated precipitation time) has been associated with increased all-cause mortality in pre-dialysis CKD [74]. In CKD stage G2–G4 patients, a lower T50 score was significantly associated with atherosclerotic cardiovascular disease events, end-stage kidney disease, and all-cause mortality, but the association was not independent of kidney function (Chronic Renal Insufficiency Cohort study) [75]. In haemodialysis patients, associations between lower T50 and higher risk of death, myocardial infarction, and peripheral vascular events are also observed (EVOLVE study) [76]. Further prospective interventional studies are needed to determine whether these associations can be causally linked.

Given the lack of PTH target levels in non-dialysis CKD, treatment modifications in clinical practice are largely based on the wanted or unwanted effects of vitamin D substitution (normo-, hypo-, hyper-calcaemia and -phosphataemia). As stated above, consecutive measurements of bone densitometry might indicate a trend in changing bone morphology which may prompt a change in treatment but are no substitute for histological diagnosis.

### What levels of vitamin D should we be aiming to achieve in patients with SHPT in non-dialysis CKD?

Guidelines have suggested that vitamin D deficiency and insufficiency be corrected using treatment strategies recommended for the general population [7, 9]. However, recent studies suggest that higher levels of 25(OH)D—exceeding those generally recommended for the general population—may be needed to control PTH in non-dialysis CKD patients [60, 77]. In a cross-sectional analysis of 14,289 unselected patients with CKD, in CKD stages G3–G5, progressively higher 25(OH)D pentiles contained progressively lower mean PTH levels with no evidence of a decreasing effect of 25(OH)D to lower PTH until 25(OH)D levels of 42–48 ng/mL (105–120 nmol/L) [77]. Progressively higher 25(OH)D concentrations were not associated with increased rates of hypercalcaemia or hyperphosphataemia. This suggests that currently recommended 25(OH)D levels (generally > 30 ng/mL) may be too low as a target for treating SPHT in CKD [77]. Further support for a higher target level comes from a post-hoc analysis of ERC Phase 3 trials, which suggested that mean 25(OH)D levels of ≥ 50.8 ng/mL are required for reductions in PTH and bone turnover markers in CKD stage G3–G4 [60]. In addition, the VITALE study demonstrated that higher levels of 25(OH)D [43.1 (12.8) ng/mL] lowered PTH and reduced fracture risk in kidney transplant patients with 25(OH)D insufficiency compared with lower levels of 25(OH)D [25.1 (7.4) ng/mL] [78]. As noted in the discussion of vitamin D metabolism earlier in this manuscript, extra-renal activation of 25(OH)D to 1,25(OH)_2_D may play an important role in active vitamin D production among CKD patients in whom renal function is impaired [35, 40]; however, this depends on adequate circulating levels of 25(OH)D and may require levels well above those traditionally considered to represent ‘sufficiency’ in the general population [60].

Several professional organisations have provided recommendations for diagnostic thresholds within their guidelines. The most widely recognised and commonly cited clinical threshold for serum 25(OH)D ‘sufficiency’ in the general population is > 30 ng/mL (> 75 nmol/L) [43]. This threshold is based on studies in which PTH levels were maximally suppressed by vitamin D supplementation, but it should be noted that none of these studies included patients with CKD. The US Institute of Medicine expert committee noted in their 2011 report that people are at risk of vitamin D deficiency at serum 25(OH)D concentrations < 12 ng/mL (30 nmol/L) and some are potentially at risk for inadequacy at levels ranging from 12 to 20 ng/mL (30–50 nmol/L) in the general population, but commented that these levels could not necessarily be extended to disease states such as CKD [34, 79]. The range of 30–100 ng/mL (75–250 nmol/L) for 25(OH)D sufficiency is cited by the Endocrine Society based on studies in various populations, with a threshold of 100–150 ng/mL (250–375 nmol/L) suggested based on safety concerns [28]. A recent consensus statement from the 2nd International Conference on Controversies in Vitamin D states that existing data are insufficient to define ‘low’ or ‘high’ vitamin D status thresholds [80]. However, despite incomplete knowledge of the role of vitamin D in many target tissues, serum 25(OH)D concentrations < 20 ng/ml (50 nmol/L) are likely to have adverse effects on health [80]. Supplemental vitamin D was shown to have a protective effect (e.g., on bone mineral density and arterial function) in patients with vitamin D insufficiency (defined as serum 25(OH)D levels < 50 nmol/L) in the ViDA study [81], whereas vitamin D supplementation had no impact on healthy adults in the VITAL study [82]. However, neither study included patients with vitamin D deficiency or at levels of insufficiency commonly seen in patients with CKD. This further suggests that vitamin D guidelines based on the general population may not be applicable to patients with vitamin D insufficiency (such as those with CKD). The 2009 KDIGO guidelines have also noted previous discussions exploring whether definitions of vitamin D sufficiency may be linked to an adequate response in PTH, and the ranges at which there is no further reciprocal reduction in serum PTH upon vitamin D supplementation [7]. Indeed, the post-hoc analyses of ERC Phase 3 trials suggested that reductions in PTH may start to attenuate above 25(OH)D levels of 50.8 ng/mL [60]. The National Kidney Foundation (NKF) Statement from 2018 states that 25(OH)D levels of 20–50 ng/mL represent a ‘modest target’ and that ‘adequacy’ is defined as ‘no evidence of counter-regulatory hormone activity’. They also note that 25(OH)D levels > 30 ng/mL might be required for extra-renal 1,25(OH)_2_D generation [79].

The question then arises as to whether there are any safety concerns associated with raising vitamin D levels to above 50 ng/mL in non-dialysis CKD [60]. Is there an upper tolerability limit for vitamin D, and what is the evidence for this in a CKD versus a healthy population [60, 80]? Observational studies have noted a reverse J-shaped association of serum 25(OH)D with cardiovascular disease mortality, with the highest risk at the lowest levels [80]. There is limited evidence on the potential risks and benefits of higher vitamin D levels in the general population and in CKD. Data from the Phase 3 ERC studies showed that a gradual elevation of the mean serum total 25(OH)D with ERC to levels as high as 92.5 ng/mL over a 26-week period had no adverse effects on mean serum calcium or phosphorus [60]. In addition, there were no adverse effects on FGF-23 or eGFR and mean serum 1,25(OH)_2_D did not increase above the upper limit of normal (62 pg/mL). Extension of these studies to 52 weeks of ERC treatment also demonstrated no increased risks related to these parameters [19, 59]. Further studies are required to determine the optimal vitamin D requirement in non-dialysis CKD, and an emerging body of real-world evidence with ERC may help to inform this question.

### How do we choose a therapeutic option for SHPT in non-dialysis CKD?

With current guidelines no longer recommending the routine use of calcitriol and active vitamin D due to increased risk of hypercalcaemia, and now a growing body of evidence suggesting that the current targets for vitamin D repletion may not be generalisable to CKD (levels of 25(OH)D ≥ 50 ng/mL may be required to control PTH) [60, 77], the optimal treatment strategies for patients with SHPT in non-dialysis CKD remain to be clearly defined. Nevertheless, the associations between elevated PTH levels and morbidity and mortality [2] indicate a need for effective management of SHPT without delaying treatment until these elevations become severe and progressive in CKD stage G4–5 and at which point the benefits of using calcitriol/active vitamin D may be more balanced against the risks of hypercalcaemia [7].

While there has been much interest to explore the therapeutic potential of nutritional vitamin D, a recent meta-analysis of randomised controlled trials suggests that nutritional vitamin D supplements in non-dialysis CKD do not reliably and consistently lower PTH even at higher doses, and the average 25(OH)D levels in treated patients do not reach > 50 ng/mL in the majority of randomised controlled trials, implying a limited potential of nutritional vitamin D to reach the 25(OH)D levels suggested as needed to effectively control SHPT [17]. These findings may be explained by the complex and variable nature of nutritional vitamin D absorption, distribution and activation that may limit its potential to achieve vitamin D > 50 ng/mL and contribute to its limited ability to reduce PTH [34].

The combined data from two Phase 3 clinical trials and a subsequent extension study demonstrate that oral ERC 30 or 60 μg is effective for treating SHPT and correcting underlying vitamin D insufficiency in adult patients with CKD stage G3 or G4. ERC further produced exposure-dependent reductions in plasma PTH and bone turnover markers when mean serum total 25(OH)D ≥ 50 ng/mL, with no adverse effects on safety parameters including serum calcium and phosphate [60].

There are currently no head-to-head studies comparing the relative safety and efficacy of treatments for SHPT in CKD. Given the differences in study designs and study populations, direct comparisons between treatments cannot currently be made and comparative clinical studies are required to more clearly define the relative benefits of different approaches. Emerging data from a recent meta-analysis suggest that compared to paricalcitol, ERC is equally effective at reducing PTH in CKD stage G3–G4 but is associated with only minimal changes in serum calcium levels [83]. Similarly, a recent real-world study in CKD stage G3–G4 found that, compared to other vitamin D therapies (active vitamin D and nutritional vitamin D), ERC significantly reduced PTH and resulted in greater increases in 25(OH)D levels, without increases in serum calcium seen in patients treated with active vitamin D [62]. Differences in effectiveness with regard to PTH reduction and 25(OH)D levels, and in hypercalcaemia in these analyses are likely explained by the lack of pharmacological surges with ERC that are associated with nutritional vitamin D and active vitamin D/its analogues, and the potential benefits of avoiding negative feedback from a ‘spike’ in 25(OH)D [52]. Steady-state 25(OH)D levels were reached after 12 weeks of dosing in the pivotal studies of ERC in CKD stage G3–G4, and averaged 50–56 ng/mL with 30 μg daily, and 69–67 ng/mL with 60 μg daily, in the two studies, respectively. The levels remained stable throughout the 52-week treatment period. Gradual elevation of mean serum 25(OH)D to these levels had minimal impact on mean serum calcium, phosphorus, FGF-23 or eGFR and did not increase mean serum 1,25(OH)_2_D above the upper limit of normal (62 pg/mL) [19, 60].

While not designed as a comparative efficacy study, an ongoing open-label, Phase 4 study (NCT03588884) will investigate the effects of ERC, immediate-release calcifediol, high-dose cholecalciferol, or paricalcitol + low-dose cholecalciferol in CKD stage G3–G4 patients with SHPT and vitamin D insufficiency. The primary outcome of this study is to evaluate pharmacokinetic/pharmacodynamic profiles, but safety and efficacy will also be assessed and may provide some insights into the relative roles of the different approaches.

## Conclusions

Despite advances in our understanding, the optimal management of SHPT in non-dialysis CKD remains challenging. While there is an increasing recognition of the need to identify and treat patients with SHPT earlier in the course of the disease, target levels of PTH are unclear, as are the levels of vitamin D required to achieve PTH reduction.

Advances in treatment include the use of ERC as an additional therapeutic option. As there are currently no head-to-head studies comparing the relative safety and efficacy of treatments for SHPT in non-dialysis CKD, direct comparisons between treatments cannot currently be made. Comparative clinical studies are required to more clearly define the relative benefits of different treatment approaches, and further research possibly with novel surrogates is needed to more clearly identify their impact on hard clinical outcomes.
